# Robotic Whipple for Abnormal Pancreaticobiliary Junction With Recurrent Acute Pancreatitis: A Case Report

**DOI:** 10.7759/cureus.94456

**Published:** 2025-10-13

**Authors:** Adam Khader, Sarah-Anne Diamond, Beth Rider, Catherine Vozzo, Munazza Anis, Ronald F Wolf, Jose G Trevino, Leopoldo J Fernandez

**Affiliations:** 1 Surgical Oncology, Central Virginia VA Health Care System, Richmond, USA; 2 Surgical Oncology, Virginia Commonwealth University School of Medicine, Richmond, USA; 3 Gastroenterology, Central Virginia VA Health Care System, Richmond, USA; 4 Radiology, Central Virginia VA Health Care System, Richmond, USA; 5 Surgical Oncology, Hoag Family Cancer Institute, Newport Beach, USA

**Keywords:** ercp, pancreatic duct stones, pancreatobiliary junction anomaly, robotic whipple, spyglass

## Abstract

Pancreaticobiliary maljunction (PBM) is a congenital anomaly that predisposes patients to recurrent pancreatitis, choledocholithiasis, and an increased risk of biliary tract malignancy. In patients without biliary dilatation, standard management typically consists of cholecystectomy with endoscopic biliary decompression. However, management can be challenging in patients with refractory recurrent pancreatitis, indeterminate strictures, or progressive ductal changes.

We describe a 62-year-old woman with PBM who presented with recurrent acute pancreatitis despite undergoing cholecystectomy, biliary sphincterotomy, and multiple endoscopic retrograde cholangiopancreatography (ERCP) interventions, including stent placements, choledocholithiasis extraction, and SpyGlass-directed biopsies, all of which were negative for malignancy. Over three years, she experienced multiple admissions for pancreatitis, progressive dilation of the common bile duct, and a persistent distal common bile duct stricture. Owing to ongoing symptoms and concern for occult malignancy, she underwent robotic-assisted pancreatoduodenectomy. Intraoperatively, bile-stained stones were identified in the pancreatic duct. Final pathology demonstrated chronic pancreatitis and fibrosis without malignancy. Her postoperative recovery was uneventful, with discharge on postoperative day four and complete resolution of pancreatitis symptoms at several months of follow-up.

This case illustrates a rare scenario of PBM-associated recurrent pancreatitis refractory to cholecystectomy and ERCP decompression. The presence of a distal intrapancreatic stricture likely contributed to ongoing symptoms, and the potential for occult malignancy remained despite multiple negative endoscopic biopsies. Robotic pancreatoduodenectomy provided both diagnostic and therapeutic benefits, permitting en bloc resection of the stricture and associated bile duct. Contemporary evidence suggests that robotic pancreatoduodenectomy offers perioperative advantages over open surgery, including reduced blood loss, fewer wound infections, and shorter hospital stays, albeit with longer operative times. These findings align with the favorable course in our patient, who recovered rapidly and remains symptom-free.

Robotic pancreatoduodenectomy can be considered a definitive treatment option in select patients with PBM and refractory recurrent pancreatitis, especially in the presence of indeterminate strictures. This approach provides both symptom relief and diagnostic certainty, while offering perioperative benefits compared with open surgery.

## Introduction

Pancreaticobiliary maljunction (PBM) is a congenital anomaly in which the pancreatic and bile ducts unite outside the duodenal wall, as defined by the Japanese Study Group on Pancreaticobiliary Maljunction [1]. This anatomical variation results in a long common channel beyond the influence of the sphincter of Oddi, allowing bidirectional reflux of bile and pancreatic juice. Such reflux can lead to impaired drainage, stone formation, recurrent pancreatitis, and malignant transformation in the biliary tract and pancreas [1,2].

Several classification systems have been proposed for PBM, including the Komi and Todani systems; however, the scheme adopted by the Japanese Study Group, based on the presence or absence of biliary dilation, offers the most clinically relevant framework [1]. The risk of malignancy in PBM is well-established, with gallbladder and bile duct cancers occurring in up to 21.6% of patients with biliary dilatation and 42.4% of those without [1,3]. Gallbladder cancer represents the majority of biliary malignancies in patients without biliary dilation [1]. Consequently, resection of the extrahepatic bile duct and cholecystectomy is generally recommended in the dilated group. In contrast, management in the absence of biliary dilation is more nuanced, often limited to cholecystectomy alone [4].

Although malignancy dominates the literature, the most common symptomatic presentation of PBM is acute pancreatitis, occurring in over 30% of cases [5,6]. In nondilated PBM, this is typically addressed with cholecystectomy and biliary decompression via endoscopic retrograde cholangiopancreatography (ERCP) and sphincterotomy [5]. However, in rare cases, symptoms persist despite intervention [5].

Herein, we present a case of a 62-year-old female with PBM and recurrent acute pancreatitis unresponsive to standard therapy, and an indeterminate biliary stricture ultimately requiring robotic-assisted pancreatoduodenectomy. Robotic pancreaticoduodenectomy has been associated with decreased blood loss, wound complications, and a shorter hospital length of stay, despite longer operative times compared to open surgery [7-9]. This report highlights the role of robotic surgery in addressing complex pancreaticobiliary anatomy and discusses the diagnostic and operative strategies involved.

## Case presentation

A 62-year-old African American female with a history of right-sided breast cancer status post mastectomy and chemotherapy in 2003, gastroesophageal reflux disease, and osteoarthritis, presented with recurrent acute pancreatitis following laparoscopic cholecystectomy for acute cholecystitis in March 2021, with pathologic findings of cholelithiasis. Her postoperative course was complicated by ascending cholangitis due to a biliary stricture, requiring biliary sphincterotomy and stent placement. Over the following three years, the patient experienced at least five documented admissions for acute pancreatitis, with lipase levels exceeding 5000 IU/L, and many more episodes managed at home, with symptoms occurring up to five days each month. She reported upper abdominal pain radiating across the epigastrium, often triggered by eating, both fatty and non-fatty foods, resulting in significant food aversion and unintentional weight loss.

**Figure 1:**
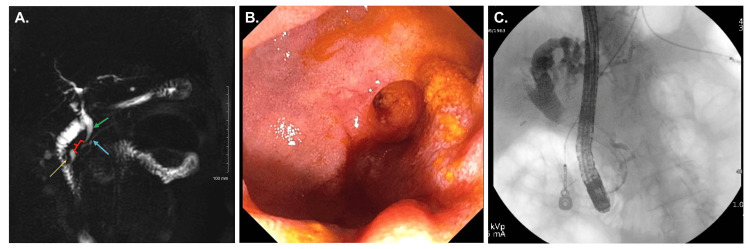
(A) Magnetic resonance cholangiopancreatography (MRCP) image showing common bile duct (green arrow) and pancreatic duct (blue arrow) forming a 1.4 cm common channel (red bracket), emptying into a duodenal diverticulum (yellow arrow). (B) Picture of an ampulla within the diverticulum. (C) Endoscopic cholangiopancreatography image demonstrating pancreatic duct backfilling with the balloon inflated in the distal common bile duct, suspicious for biliary-pancreatic reflux.

**Figure 2:**
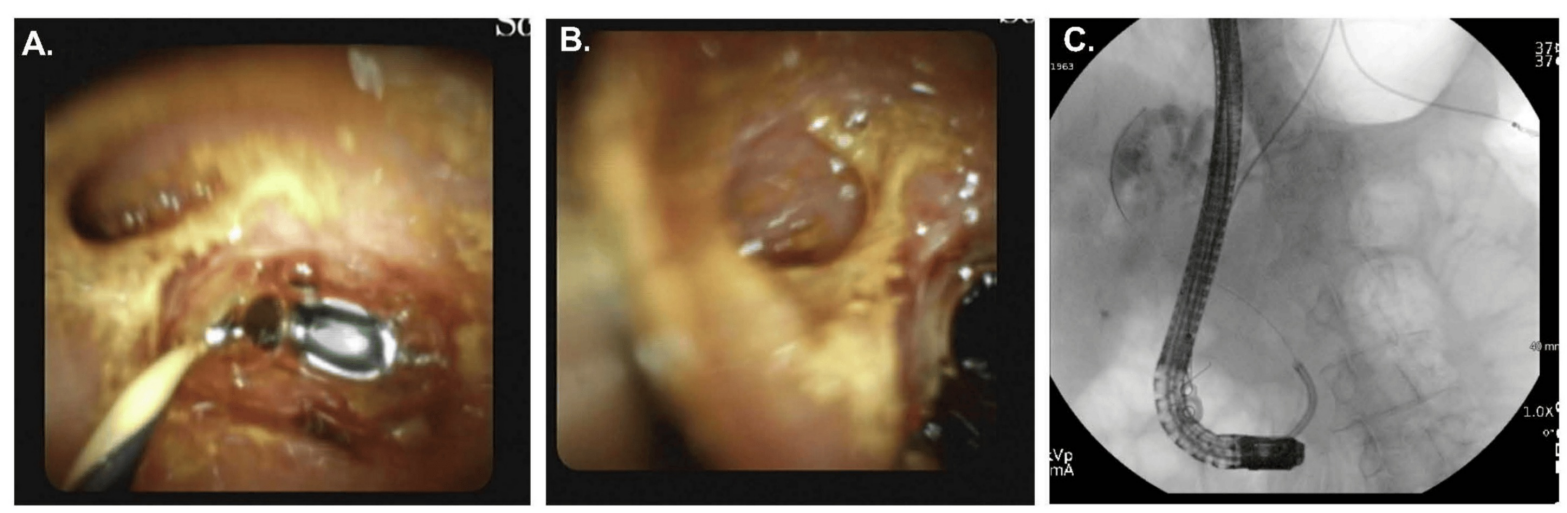
Spyglass images showing (A) the wire in the common bile duct and pancreatic duct take off at 10 o'clock. (B) Pancreatic duct in focus. (C) Fluoroscopic position of Spyglass.

A total of seven ERCPs were performed between 2021 and 2024, including biliary sphincterotomy, stent placements, choledocholithiasis extraction, needle knife papillotomy, and cholangioscopy with brushings and biopsies. Imaging consistently demonstrated a long common channel (Figure 1), choledocholithiasis, and a benign-appearing biliary stricture in the distal bile duct (Figure 2). The diameter of the common bile duct progressively dilated from 7 mm (endoscopic ultrasound (EUS), March 2024) to 13 mm (ERCP, June 2024). All cytology, Spybite biopsies, and brushings were negative for malignancy (Figure 2). Autoimmune pancreatitis was considered unlikely given normal levels of immunoglobulin G4 (IgG4). Despite temporary symptom relief following biliary decompression, the patient continued to experience recurrent pancreatitis. Following a final ERCP with stent placement in June 2024, she experienced clinical improvement but remained interested in definitive surgical management due to the need for periodic stent exchanges.

**Figure 3:**
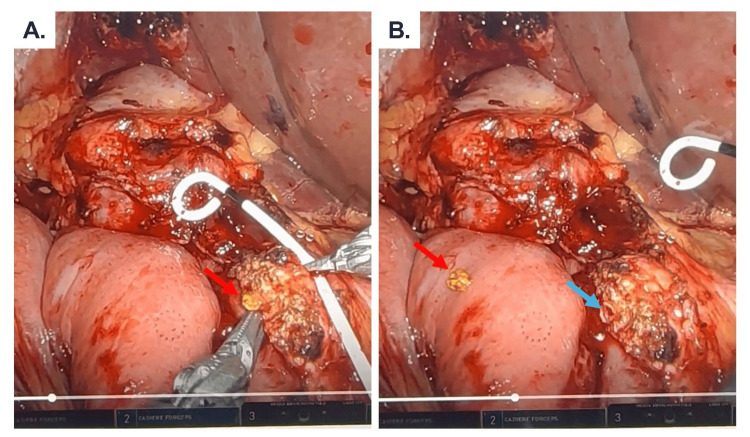
Intraoperative picture of a bile-stained stone (red arrow) in the pancreatic duct, pathognomonic of biliary-pancreatic reflux seen during the pancreatic transection portion of the robotic pancreaticoduodenectomy. Pancreatic duct (blue arrow).

In March 2025, she underwent an uneventful robotic-assisted pancreatoduodenectomy. Intraoperatively, pathognomonic bile-stained stones were detected in the pancreatic duct during pancreatic neck transection (Figure 3). Surgery duration was 323 minutes, and estimated blood loss was less than 50 mL. Final pathology revealed chronic pancreatitis with parenchymal fibrosis, diffuse necrosis, an obstructed indwelling bile duct stent filled with stones, and no evidence of malignancy. Her postoperative course was uneventful. Drain amylase level was 129 IU/L on postoperative day three, prompting drain removal, and she was discharged on postoperative day four. At her most recent follow-up in October 2025, the patient remained symptom-free, with no further episodes of abdominal pain or pancreatitis, and reported gradual improvement in dietary intake and overall activity level - an excellent clinical outcome.

## Discussion

This case highlights an unusual presentation of persistent acute pancreatitis in a patient with PBM despite undergoing cholecystectomy and biliary sphincterotomy, interventions generally considered standard treatment in the absence of bile duct dilation [4,5]. The patient’s anatomy was further complicated by a major papilla located within a duodenal diverticulum and a persistent distal common bile duct (CBD) stricture extending into the intrapancreatic portion of the duct. Ultimately, definitive symptom resolution was achieved through robotic-assisted pancreatoduodenectomy, a procedure not commonly performed for PBM.

In most cases of PBM without biliary dilation, cholecystectomy followed by biliary decompression via ERCP and sphincterotomy is sufficient to reduce the frequency and severity of pancreatitis. However, our patient continued to experience frequent attacks despite these interventions. One possible explanation is that the stricture within the common channel prevented adequate decompression of the biliary system, thereby sustaining the abnormal reflux between bile and pancreatic secretions, a hallmark of PBM pathophysiology. While such a mechanism is speculative, similar cases of post-sphincterotomy recurrent pancreatitis in PBM have been described [5], though long-term follow-up and definitive management with surgery remain uncommon in the literature.

An important consideration in this case was the risk of malignancy. According to a nationwide survey in Japan (n = 1511 adult patients), one-third of patients with PBM were not associated with biliary dilation. Biliary cancer was detected in 21.6% of adult patients with congenital biliary dilatation and in 42.4% of PBM patients without biliary dilatation. There was a predominance of gall bladder cancers (88.1%) compared to bile duct (7.3%) or gallbladder and bile duct cancers (4.1%). The rate of pancreatic cancer was 1% [3]. In our patient, there was no clear biliary dilation prior to cholecystectomy, but she subsequently developed progressive dilation of the CBD. In addition, the presence of an indeterminate distal CBD stricture warranted concern. Despite multiple negative brushings and biopsies, the possibility of occult malignancy could not be excluded, particularly in the setting of worsening symptoms, progressive ductal changes, and chronic inflammation.

The risk of malignancy in patients with biliary strictures alone is high. According to the American College of Gastroenterology (ACG) clinical guideline, the prevalence of malignancy among patients with obstructive jaundice undergoing endoscopic ultrasound-guided fine-needle aspiration (EUS-FNA) is approximately 73%, with overall rates of cancer in suspected malignant strictures ranging from 74% to 87%, and as high as 80-95% in patients taken to surgery for a suspected malignant stricture [10]. If we consider that our patient did not present with jaundice and significant laboratory abnormalities, the likelihood of malignant stricture remains clinically significant but is lower than is typically reported. For instance, in a recent retrospective cohort, malignancy was present in 75.8% of patients with jaundice, but still occurred in 38.5% of those without jaundice [11]. A recent meta-analysis revealed that two-thirds of patients with indeterminate brushing cytology were later diagnosed with malignancy on follow-up or through histopathology [12]. At our institution, we perform Spy-Glass-directed biopsies, which enable the collection of larger samples directly from the stricture area (Figure 2). The rationale being that it would decrease our false negative results. In another meta-analysis of 11 studies involving 323 patients, SpyBite biopsy during digital single-operator cholangioscopy demonstrated a sensitivity of 74% (95% CI: 67-80%) and a specificity of 98% (95% CI: 95-99%), with a pooled negative likelihood ratio of 0.34 (95% CI: 0.26-0.44) and a diagnostic odds ratio of 35.3 (95% CI: 17.4-71.4). These results indicate that while a positive SpyBite biopsy is highly predictive of malignancy, the false-negative rate remains substantial, and a negative result cannot definitively exclude cancer [13].

Given the intrapancreatic location of the stricture and the limitations of endoscopic management, the decision was made to proceed with pancreatoduodenectomy. Although biliary bypass or segmental bile duct resection might be appropriate for more proximal strictures, these approaches risk leaving behind an occult malignancy. Pancreatoduodenectomy offered both therapeutic and diagnostic benefits, with complete removal of the involved intrapancreatic bile duct segment and pathologic evaluation confirming chronic pancreatitis and intraductal stones.

The use of robotic surgery in this case provided technical advantages, including enhanced visualization of complex biliary anatomy and precise dissection around inflamed and fibrotic tissues. Robotic pancreatoduodenectomy, though technically demanding, has been shown to offer perioperative benefits over open and laparoscopic approaches. In a 2019 meta-analysis, robotic pancreaticoduodenectomy was associated with significantly less blood loss (weighted mean difference (WMD): −374 mL, 95% CI: −506.8 to −241.2), shorter hospital stay (WMD: −5.2 days, 95% CI: −8.4 to −1.9), and lower wound infection rates (OR: 0.17, 95% CI: 0.04-0.80), at the cost of longer operative time (WMD: +71.7 minutes, 95% CI: 23.4-120.1) [7]. Kabir et al. further demonstrated that robotic pancreaticoduodenectomy had less blood loss than both laparoscopic (mean difference (MD): −112.6 mL, 95% CI: −36.9 to −118.2) and open procedures (MD: −209.9 mL, 95% CI: −140.4 to −279.4), with reduced delayed gastric emptying (OR: 0.59, 95% CI: 0.39-0.90 vs. OPD) and fewer wound infections (OR: 0.35, 95% CI: 0.18-0.71 vs. OPD) [8]. In a 2024 nationwide propensity-matched study, robotic pancreaticoduodenectomy was again associated with less blood loss (200 vs. 500 mL, p < 0.001), fewer wound infections (7.4% vs. 12.2%, OR: 0.57, p = 0.008), and shorter hospital stay (11 vs. 12 days, WMD: −1.0 day, p < 0.001), though with longer operative times (359 vs. 301 minutes, WMD: +58 minutes, p < 0.001) [9]. Our patient experienced an uneventful recovery, was discharged on postoperative day four, and has remained free of abdominal pain or pancreatitis at seven months of follow-up. While we do not have specific recommendations for this benign entity, we perform lifetime surveillance of post-pancreaticoduodenectomy patients on an annual basis to screen for nutritional deficiencies and late complications [14].

## Conclusions

In the absence of bile duct dilation, surgical treatment of recurrent acute pancreatitis in patients with PBM is controversial, with many patients responding to cholecystectomy and biliary decompression with ERCP sphincterotomy. In cases refractory to ERCP interventions, surgical treatment can be considered. The decision for biliary diversion versus resection can be complicated and must consider the risk of malignancy and patient comorbidities. Minimally invasive pancreaticoduodenectomy can be an effective treatment option in the appropriate clinical setting and in centers where the technology and expertise are available. As this is a single-case report, broader conclusions should be drawn cautiously, and further studies or case series are needed to better define the role of minimally invasive pancreaticoduodenectomy in this patient population.
